# A Task Scheduling Optimization Method for Vehicles Serving as Obstacles in Mobile Edge Computing Based IoV Systems

**DOI:** 10.3390/e25010139

**Published:** 2023-01-10

**Authors:** Mingwei Feng, Haiqing Yao, Jie Li

**Affiliations:** Institute of Logistics Science and Engineering, Shanghai Maritime University, Shanghai 201306, China

**Keywords:** vehicle-to-infrastructure, internet of vehicles, mobile edge computing, channel occlusion, task scheduling, non-dominated sorting genetic algorithm-III

## Abstract

In recent years, as more and more vehicles request service from roadside units (RSU), the vehicle-to-infrastructure (V2I) communication links are under tremendous pressure. This paper first proposes a dynamic dense traffic flow model under the condition of fading channel. Based on this, the reliability is redefined according to the real-time location information of vehicles. The on-board units (OBU) migrate intensive computing tasks to the appropriate RSU to optimize the execution time and calculating cost at the same time. In addition, competitive delay is introduced into the model of execution time, which can describe the channel resource contention and data conflict in dynamic scenes of the internet of vehicles (IoV). Next, the task scheduling for RSU is formulated as a multi-objective optimization problem. In order to solve the problem, a task scheduling algorithm based on a reliability constraint (TSARC) is proposed to select the optimal RSU for task transmission. When compared with the genetic algorithm (GA), there are some improvements of TSARC: first, the quick non-dominated sorting is applied to layer the population and reduce the complexity. Second, the elite strategy is introduced with an excellent nonlinear optimization ability, which ensures the diversity of optimal individuals and provides different preference choices for passengers. Third, the reference point mechanism is introduced to reserve the individuals that are non-dominated and close to reference points. TSARC’s Pareto based multi-objective optimization can comprehensively measure the overall state of the system and flexibly schedule system resources. Furthermore, it overcomes the defects of the GA method, such as the determination of the linear weight value, the non-uniformity of dimensions among objectives, and poor robustness. Finally, numerical simulation results based on the British Highway Traffic Flow Data Set show that the TSARC performs better scalability and efficiency than other methods with different numbers of tasks and traffic flow densities, which verifies the previous theoretical derivation.

## 1. Introduction

On-board applications become increasingly diverse with the rapid development of IoV, which requires transmitting a large amount of information through communication links. These workflow applications, such as automatic driving, voice recognition and entertainment information, usually require intensive computing resources [1]. However, a single on-board unit (OBU) may not be able to provide sufficient computational resources, and it is difficult to satisfy the users’ high quality of service (QoS). Mobile edge computing (MEC) provides a feasible scheme for the above problems [2]. The edge equipment has a certain computing power, which can realize computing force sinking on the RSU and reduce the pressure of cloud servers. In addition, it can reduce the consumption of time in the network, simplify the network structure, and achieve an accurate perception of the traffic status [2]. Therefore, mobile edge computing has obvious advantages in the construction of smart roads, especially in the IoV systems. After introducing MEC into the IoV systems, these edge computing devices can directly receive local data in time from the OBU and transmit it to the RSU in the adjacent area with a low delay, and the whole process can be completed in milliseconds. Furthermore, the dense dynamic traffic flow on the middle lane will lead to an occlusion effect on the wireless link in the V2I communication. Occlusion refers to the phenomenon that obstacles block the wireless link, which may lead to the attenuation of signal strength. In contrast to the traditional edge computing system, the transmission rate, interference, and energy consumption will be extremely affected by the dynamic occlusion [3]. In addition, due to the redundancy coverage problem caused by the full coverage model of RSU [4], mobile vehicles with high speed may have diverse upload paths at any time. Therefore, it is significant to propose an efficient and suitable scheduling method in the case of dynamic dense traffic flow.

Task scheduling strategies under vehicle-road-cloud intelligent collaboration have aroused broad interest of some scholars in recent years. A lot of existing work mainly focuses on optimizing a single factor [5,6], such as delay or energy, etc. At the same time, some research combines them and proposes multi-objective optimization methods [7]. However, most of them ignore the impact of dynamic occlusion [8,9], which will attenuate the wireless signal, thus further affecting the scheduling strategy of the application layer. Even though some scholars take the occlusion factor into consideration in the research of link scheduling [10], they regard the obstacle as a metal shield, and do not conduct a deterministic quantitative modeling for the occlusion. Based on these efforts, some scholars further consider the occlusion effect of static objects, such as trees, buildings and other large infrastructures, on wireless links. However, rare work considers the occlusion of mobile vehicles as obstacles, especially in an environment with dynamic dense traffic flow. When the orientation and position of the obstacle vehicles on the middle lane change, the occlusion degree of the wireless link will also change at the same time. These available models may not accurately depict the degree of occlusion, so they are not suitable for the scenes we proposed in this paper. In addition, the instability of the wireless link between the mobile OBU and the corresponding RSU is aggravated by the nonlinear change of traffic flow, which subverts the existing task scheduling method based on the deterministic model, making transmission unreliable and consumption unpredictable [11]. Moreover, the task scheduling problem with regard to dynamic dense traffic flow occlusion has not been considered before. Above all, some methods are proposed by us to solve the problems of dynamic dense traffic occlusion and the diversity of upload paths under redundant coverage in task scheduling. Firstly, a scene with dynamic dense traffic flow is built based on the RSU full coverage model [12]. Next, the reliability is redefined, and two reliability constrained task scheduling methods are proposed to simultaneously optimize the execution time and calculating cost of each workflow application. The contributions of this paper are listed as follows:

(1) The dynamic dense traffic flow model considering the occlusion impact on the V2I communication link is proposed based on the Huygens–Fresnel theory.

(2) A multi-objective optimization task scheduling problem for optimizing both execution time and calculating cost with the constraint of reliability is proposed, where competitive delay is introduced to describe the situation of channel resource contention, and the reliability constraint is proposed based on the average packet loss rate, which is calculated according to the real-time occlusion on the V2I communication.

(3) The approximate solution method named TSARC pays attention to the reservation of the boundary solution when compared with GA, which has better performance in solving the extremely convex problem, and the part of the numerical simulation and analysis verifies the efficiency and scalability of these methods. 

The rest of this paper can be organized as follows: related work is given in Section 2. The description of problems and models of the system are given out in Section 3. Next, Section 4 gives two approximate solutions. Numerical results in simulations and real scenes are shown in Section 5. Section 6 concludes this paper and gives the direction of the future research.

## 2. Related Work

In spite of the fact that there is no current approach for task scheduling in the scenario we propose, the efforts of some scholars have inspired our study. The high-speed movement of vehicles has led to frequent changes in the topologies of the vehicle networks, unstable connections between vehicles, and highly dynamic properties of channel models. These characteristics have prompted lots of scholars to focus on ensuring the QoS of various services in the IoV systems, such as delay, throughput, reliability, etc. In [13], Keshari et al. investigate various problems that vehicular fog computing (VFC) faces while allocating resources and retrieving data, such as operations for dynamic topology management and high mobility of vehicles, etc. This results in inhomogeneous resource distribution of tasks due to inefficient resource allocation and delayed data delivery due to impractical data retrieval. Additionally, the security and privacy of traffic data are threatened because of sharing in the vehicle fog. Then they develop an in-depth investigation on each level in the VFC process in order to overcome the challenges. In order to categorize the difficulties encountered at each level, they created a new categorization system. In [2], Hou et al. propose a joint allocation of wireless resource and MEC computing resources (JAWC) algorithm to reduce the network’s overall delay and guarantee the reliability of the vehicular UE(VUE). The vehicle-to-everything (V2X) connection aggregation and MEC computing resource scheduling are the two stages of the JAWC algorithm. To create the best resource allocation matrix, a spectral radius-based interference cancellation (SR-IC) method is suggested in the first stage. The best task offloading proportion of the VUE and MEC’s computation resource allocation may be found in the second stage by converting the initial optimization issue into a convex issue. In [5], Pang et al. concern a hybrid transmission and reputation management (HTRM) system aimed at reducing delay in the smart network. An effective hybrid vehicle-to-vehicle (V2V) and V2I scheduling algorithm (EH-V2VV2I) is developed based on fifth generation mobile communications (5G) vehicle-to-everything (V2X) technology, which utilizes relay vehicles to assist initiating vehicles for task transmission. An approach called relay vehicle selection and reputation management (RV-SRM), which can predict the link survival time, select highly reliable relay cars and manage vehicle credibility at the same time, is proposed to increase the reliability of V2V connections. In [14], Kang et al. propose an adaptive transmission power and message interval control (ATPMIC) strategy for C-V2X Mode 4, in which each vehicle makes use of real-time channel sensing, as well as neighbor information to reduce the contention of the channel for improving reliability and delay. In [10], Xiao et al. proposed a decentralized forwarding-aware cooperative scheduling (FACS) method for real-time and reliable basic safety message (BSM) dissemination. In this method, broadcast links are carefully scheduled to coincide with the local resource of adjacent vehicles and RSUs, such as the assigned task in BSM recovery. Thus, the time required for the BSM dissemination may be reduced, and other vehicles would have more probability to forward BSMs for real-time and reliable BSM dissemination.

The explosive growth of access demand for IoV has intensified the fierce competition between vehicles for limited resources, such as the computing resource and bandwidth resource. Basically, how to realize efficient resource utilization is a major challenge for task scheduling. In [15], for the purpose of scheduling data flows of unmanned aerial vehicles (UAV) effectively within the swarm, Mishra et al. conceive a unique, cooperative multi-hop communication model based on the C-V2X (cellular vehicle-to-everything) Mode 4 cellular sidelink (PC5) radio interface. The model design envisions a new cellular UAV-to-everything (C-U2X) communication paradigm by optimizing cellular communications on UAV-to-UAV (U2U) and UAV-to-infrastructure (U2I) connections by using a unique interference-aware scheduling method. The centralized version of the issue is solved via an optimization model, and a distributed dynamic consensus-based bundle algorithm (D-CBBA) is suggested to provide the optimal subchannel scheduling for optimizing data transmission in a distributed environment. In [8], by introducing the unique polynomial-time heuristic algorithm named MUltiChannel Scheduler (MUCS), Naghsh et al. investigate a scheduling problem and then equals it as a vehicle routing problem to realize conflict-free scheduling in cellular V2X communications. MUCS can adapt to standard device-to-device topologies without enforcing any packet segmentation, which serve as the foundation of V2X networks. Thus, MUCS reduces the amount of control information required by the cellular V2X standard. However, the 3GPP standards do not provide any specified resource scheduling plan to take resource allocation under Mode 3 when compared with Mode 4. In [16], Sempere-Garcia et al. introduce a scheduling method based on context for LTE-V2X Mode 3 that make use of the geographical location of the vehicles and provides a dynamic configuration of its operation with the goal that all vehicles encounter an equivalent level of interference when resources need to be shared. In [6], He et al. propose a short-term sensing-based resource selection (STS-RS) strategy to minimize packet collisions caused by contention for resources. Before the selection of resources, there is a short-term sensing duration configuration at the start of the resource unit right. In addition, the result of sensing determines whether the packet is finally transferring to the chosen resource. Moreover, they also deduce analysis models about the performance for the STS-RS method and SPS method mentioned in C-V2X Mode 4. However, the above task scheduling methods omit the occlusion effect of obstacles. 

Some scholars take into account the occlusion effect in the V2V or V2I communication. Buildings and other structures in metropolitan areas may block the line of sight between the OBU and RSU, thus narrowing the vehicular communication range and affecting the effectiveness of traffic safety applications negatively, particularly at intersections. Thus, some scholars consider the influence of these static obstacles. In [17], Kim et al. concentrate on the improvement of urban V2V communications at intersections by using a transmission technique that adapts to the environment. The impacts of shadowing on urban V2V communication at intersections may be eliminated by using an adaptive beamforming scheme based on neuroevolutionary augmentation topologies to regulate the radiation pattern of an antenna array. In [18], Selvakumari et al. propose the Chew’s first Delaunay triangulation refinement system (CFDTRS) for the deployment of RSUs to provide optimum network coverage in the vehicular network with the minimum cost. When determining where to deploy RSUs, CFDTRS takes into consideration the following variables: the density of traffic on the map, the quantity of obstructions, the intersection popularity, and the coverage ratio. In order to obtain maximum coverage in the convex map and ensure that each region in the convex map is fully covered by at least one RSU in the presence of multiple barriers, it is recommended to deploy the proper number of RSUs within the range of data transmission. As for the scenario of vehicles serving as obstacles, the current research involves the case of a static single vehicle as an obstacle, and a few of them discuss the dynamic dense traffic flow. In [19], Turner et al. utilize the V2I propagation model to conduct a signal attenuation analysis caused by the presence of metal objects in low density over an obstacle-free environment on an actual parking lot. Two scenarios are evaluated by them, which includes LOS and NLOS conditions with static metal objects, such as obstacle-free, cars, and buses. The purpose of this study is to compare the signal strength induced by metal blockage on radio wave propagation on the presence of vehicles as an obstruction to an environment without any obstacles. To verify the effectiveness of the obtained measurements, the received signal strength indicator (RSSI) and approximation analysis (RMSE) are used to show the validity of the data. In [20], Boban et al. measure V2V channels in four distinctive frequency bands under urban and highway conditions to discuss the impacts of vehicle blockage. They concern the impact of the frequency dependence of these parameters on the received power and fast fading characteristics under blockage, as well as the impact of the blocker size and location. Based on the results, they conclude that the blockage loss and the angular/delay spread are significantly affected by the size of the blocking vehicle. The blocker’s location in relation to the transmitter and receiver is also crucial. On the other hand, the blocking loss and the number of scattered MPCs both vary slightly with the growth of frequency, thus the frequency independence is limited. These efforts provide a theoretical basis and inspiration for our work, but none of them can be applied to our problem directly. First, based on the Huygens–Fresnel principle and diffraction theory, we propose a dense traffic flow model with vehicles serving as obstacles. Second, subject to the traffic flow model, we introduce the execution time and calculation cost model, and then a multi-objective optimization scheduling problem under the constraint of reliability is proposed, which is not found in previous work. Finally, with regard to the NP-hardness of this novel problem, approximate solutions with controllable performances, such as accuracy and efficiency, have to be proposed, which are also new challenges.

## 3. Corresponding Models and Problem Formulation

### 3.1. Model of Dynamic Dense Traffic Flow

**Figure 1:**
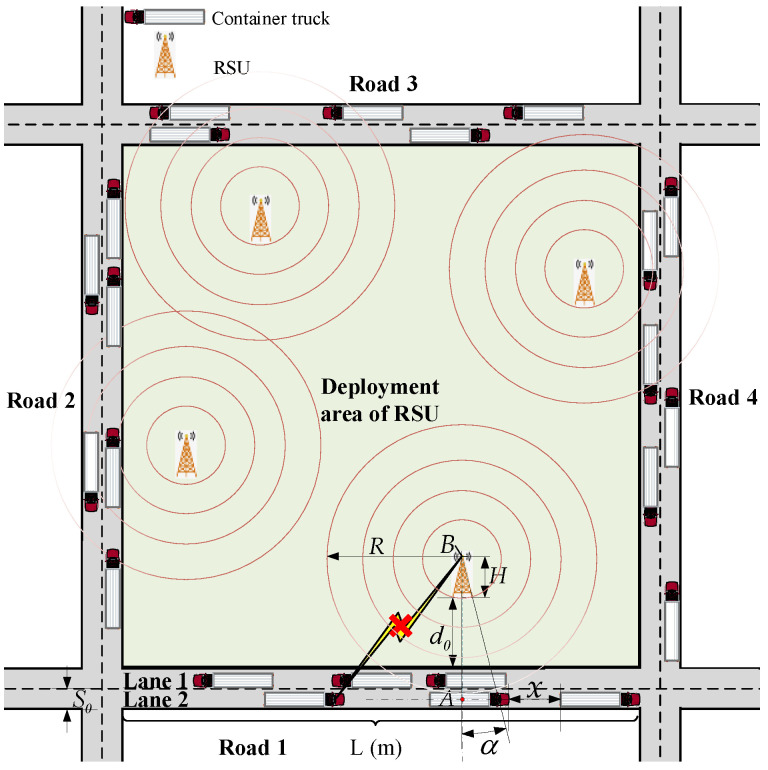
The dynamic dense traffic flow model under the condition of fading channel.

Based on the channel model proposed in our previous work [12], the scene of dynamic dense traffic flow is introduced, as shown in Figure 1, which is typical of highways, city roads, or smart ports. In addition, a large number of container trucks are driving on the roads with their dimensions adopted for normalization. Furthermore, all trucks are supposed to drive along the middle lines of the two-way lanes on account of the negligible lane width when compared with the transmission distance between the OBU and RSU.

In the above scene, the arrival process of trucks can be modeled as a Poisson process. Thus, the spacing probability density function (PDF) of any two adjacent trucks can be obtained as follows [21]:

(1) $$P\left(N=k\right)=\frac{\left(\gamma L{)}^{k}\right.}{k!}e^{-\lambda L}$$

where *γ* represents the average arrival rate of the Poisson process, which equals the density of the traffic flow, and *x* is the distance between any two adjacent trucks, as shown in Figure 1.

**Figure 2:**
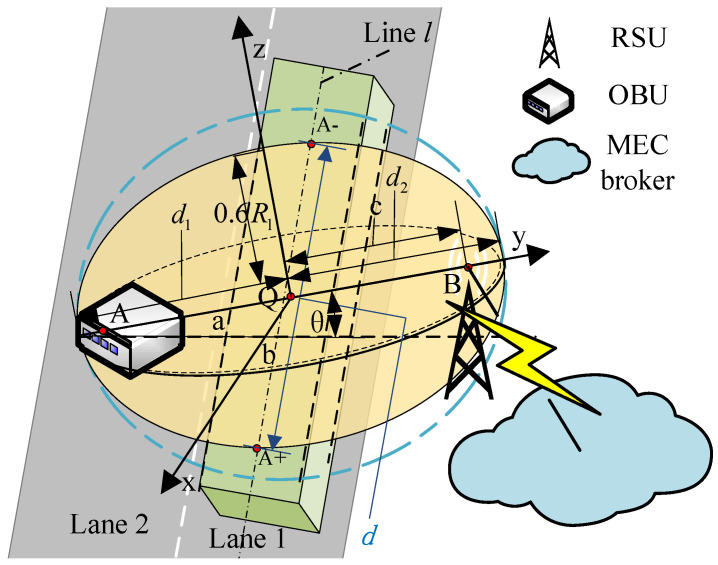
The schematic diagram of traffic flow occlusion on a V2I communication link.

Next, the NLOS and LOS conditions are distinguished based on the Huygens–Fresnel theory. The Huygens-Fresnel theory is the theoretical basis for studying diffraction phenomena, and it can be used as an approximate method to solve wave (especially light wave) propagation problems. The intermediate space between the OBU and RSU can be subdivided into a cluster of Fresnel ellipsoids, which is used to analyze the propagation of radio waves. Any obstruction hindering the first Fresnel ellipsoid may have a negative impact on the propagation of signals, according to the theory of electromagnetic wave propagation. Generally speaking, the effects of diffraction may be ignored and a LOS communication connection is established when the intrusion area of obstacles is less than 60% of the first Fresnel ellipsoid [22]. Otherwise, it is an NLOS link. Figure 2 shows the schematic diagram of traffic flow occlusion on a V2I communication link.

When the line linking the OBU with the RSU is perpendicular to the middle line of lane 1, the radius of the first Fresnel ellipsoid between A (the apex of the OBU) and B (the apex of the RSU) can be calculated as follows [23]:

(2) $$R_{1}=550{\left[\frac{d_{1}d_{2}}{\left(d_{1}+d_{2}\right)f}\right]}^{\frac{1}{2}}$$

where *f* denotes the frequency of the V2X signal, and $d_{1}$ and $d_{2}$ denote the distances between *A* and *B* at *Q* where the first Fresnel ellipsoid radius is calculated.

The distance between the intersection points of line *l* and ellipsoid *E* is regarded as the occlusion length of traffic flow. $A_{+}(x_{+},y_{+},z_{+}),A_{-}(x_{-},y_{-},z_{-})$ are the coordinates of the two intersection points, the direction vector of *l* is represented as $\vec{l}(\cos\alpha ,\sin\alpha ,0)$, and *l* passes through $Q(x_{0},y_{0},z_{0})$. On the basis of our previous research, the length of $A_{+}A_{-}$ can be calculated as follows [12]:

(3) $$d=\sqrt{{\left(y_{+}-y_{-}\right)}^{2}+{\left(x_{+}-x_{-}\right)}^{2}}$$

According to Equation (1), the probability of NLOS can be calculated as:

(4) $$P_{\mathrm{NLOS}\;}=\int_{0}^{d}\gamma e^{-\gamma x}dx$$

Additionally, the probability of LOS can be calculated as:

(5) $$P_{\mathrm{LOS}\;}=1-P_{\mathrm{NLOS}\;}$$

For the LOS condition, the two-ray model is considered. Next, the attenuation of the signal in the free space can be represented as follows [24]:

(6) $$PL={\left(\frac{\lambda }{4\pi }\right)}^{2}{\left|\frac{e^{-jKr_{d}}}{r_{d}}+\omega \frac{e^{-jKr_{r}}}{r_{r}}\right|}^{2}$$

where *K* is the wave quantity, *λ* is wavelength, $r_{d}$ equals to the direct distance between OBU and the corresponding RSU, $r_{r}$ represents the distance reflected on the ground and *ω* represents the coefficient of reflection. Referring to the model of vertical polarized antenna [20], *ω* can be denoted as:

(7) $$\omega =\frac{\sin\theta_{1}-\frac{1}{\varepsilon }\sqrt{\varepsilon -\cos^{2}\theta_{1}}}{\sin\theta_{1}+\frac{1}{\varepsilon }\sqrt{\varepsilon +\cos^{2}\theta_{1}}}$$

where $\theta_{1}$ represents the incident angle of a reflected ray via the ground, and ε represents relative permittivity.

Concerning the condition of NLOS, the approximate value of this diffraction attenuation J that is calculated with the knife-edge diffraction model is calculated as follows [25]:

(8) $$J=\left\{\begin{array}{c} 6.9+20\log_{10}\left[\sqrt{{\left(v-0.1\right)}^{2}+1}+v-0,1\right],v\geq -0.78\\ 0,\;v\leq -0.78 \end{array}\right.$$

where the Kirchhoff parameter *v* can be calculated as follows:

(9) $$v=h\sqrt{\frac{2}{\lambda }\left(\frac{1}{r_{1}}+\frac{1}{r_{2}}\right)}$$

where *h* represents the height of the obstacle above the line that connects $T_{X}$ and $R_{X}$, $r_{1}$ and $r_{2}$ represent the distances from the top of the occlusion to the $T_{X}$ or $R_{X}$, respectively.

Next, the attenuation ${\mathrm{LOSS}}_{NLOS}$ in the condition of NLOS equals to the sum of J and PL. Then, the attenuation ${\mathrm{LOSS}}_{LOS}$ in the condition of LOS only contains PL when J=0.

(10) $${\mathrm{LOSS}}_{NLOS/LOS}=PL+J$$

The received power $D_{r}$ of RSUs in the condition of NLOS or LOS is represented as follows:

(11) $$D_{r}=D_{s}-{\mathrm{LOSS}}_{NLOS/LOS}$$

where the transmitting power of OBUs is represented by $D_{S}$.

The threshold *τ* of minimum sensitivity of the received signal strength is used to denote the coverage area of RSUs. Next, the binary parameter $P_{\mathrm{nloss}}^{\mathrm{NLOS}}$ ($P_{\mathrm{nloss}}^{\mathrm{LOS}}$) determining whether to transmit successfully in the NLOS or LOS condition is represented as below:

(12) $$P_{\mathrm{nloss}}^{\mathrm{NLOS}/\mathrm{LOS}}=\left\{\begin{array}{c} 0,{\mathrm{LOSS}}_{NLOS/LOS}>D_{s}-\tau \\ 1,{\mathrm{LOSS}}_{NLOS/LOS}\leq D_{s}-\tau \end{array}\right.$$

The packet success rate $P_{\mathrm{nloss}\;}^{s}$ at any central road point *s* in lane 2 is calculated as follows:

(13) $$P_{\mathrm{nloss}\;}^{s}=P_{\mathrm{NLOS}\;}\cdot P_{\mathrm{nloss}}^{\mathrm{NLOS}}+P_{\mathrm{LOS}\;}\cdot P_{\mathrm{nloss}}^{\mathrm{LOS}}$$

Due to the complementary relation between the packet loss rate and the packet success rate, the packet loss rate at any position *s* is represented as:

(14) $$P_{\mathrm{loss}\;}^{s}=1-P_{\mathrm{nloss}\;}^{s}$$

The variables and parameters required in the problem statement are summarized in Table 1.

### 3.2. System Architecture

This system consists of *e* RSUs, *u* OBUs, and one or several MEC brokers. The MEC broker directly communicates with RSUs, which is a device with computing power comparable to RSU (idle RSU can be used to replace it with budget constraints). All requests from passengers will be immediately forwarded to the MEC broker via RSUs, which is responsible for analyzing, estimating, and scheduling all tasks to be executed in the RSU layer. These requests usually include the real-time locations of OBUs, the locations of RSUs, the number of instructions and the required resource usage, etc. MEC brokers are located on the side close to the RSU, so the time consumed by data communication between them can be ignored. Basically, cloud servers are more powerful than RSUs, but they are more expensive to use. Cloud servers are usually located in remote locations, which implies that the task scheduling process is computational and communication heavy. Therefore, MEC brokers play an important role in the edge environment as a substitute for cloud servers.

The RSUs have been deployed and achieved full coverage of the four target roads. In addition, the algorithm running on the MEC broker aims to find a task scheduling method for all RSUs with an optimal execution time and calculating cost. The steps of task scheduling are shown in Table 2:

When the information sent by OBUs is transmitted to RSUs, it is decomposed into independent tasks, which are processed on the RSU processors. The set of all tasks sent at any time is denoted as a workflow application $WA_{j}$. Furthermore, the quantity of tasks sent by each OBU is random. According to the real-time relative position information of each pair of the OBU and RSU, the contention of channel resources within the coverage area of each RSU and the packet loss rate of each wireless link can be obtained. Each task is composed of the following attributes: the source OBU’s location, instruction amount, required memory, as well as size of the input files and output files. In addition, $T_{k}$ represents the *k*th task, and the group of *n* tasks that are transmitted to the RSU system are expressed as below:

(15) $$T=\left\{T_{1},T_{2},T_{3},\ldots ,T_{n}\right\}$$

RSUs and the cloud server have similar attributes, such as the frequency of CPU, CPU utilization, memory utilization, and bandwidth limitation. In addition, because of the limitation of the propagation ability of OBUs, it is hard for them to communicate directly with the remote cloud server. The set of *e* RSUs in the system is expressed as:

(16) $$N=\left\{N_{1},N_{2},N_{3},\ldots ,N_{e}\right\}$$

where $N_{i}$ represents the ith RSU.

Each task $T_{k}$ is dispensed to the RSU $N_{i}$, which is denoted as $T_{k}^{i}$. One or more tasks can be handled by one assigned RSU:

(17) $$TaskN_{i}=\left\{T_{x}^{i},T_{y}^{i},\ldots ,T_{z}^{i}\right\}$$

The task scheduling process can be represented as a search for a set:

(18) $$TaskScheduling=\left\{T_{1}^{a},T_{2}^{b},T_{3}^{c},\ldots ,T_{n}^{z}\right\}$$

### 3.3. Execution Time Model

The execution time is defined to describe the time domain characteristics of the system, which includes competition delay, propagation delay, and processing time. It is a key parameter to evaluate the QoS of a system [26]. In this paper, the competition delay can properly describe the channel resource contention and data conflict in the dynamic dense traffic flow scene [14]. Propagation delay describes the time taken for the electromagnetic signal to travel a certain distance in the transmission medium. Furthermore, the propagation delay is calculated by the ratio of the length of the transmission medium to the propagation rate of the electromagnetic wave on the channel, which can usually be ignored [26]. Processing time refers to the total time spent on tasks to complete the whole computational procedures in the processors. 

#### 3.3.1. Competition Delay

The competition delay occurs when multiple tasks are transmitted to the same RSU. The V2I transmission adopts the mechanism named carrier sense multiple access with collision avoidance (CSMA/CA) in IEEE802.11p in order to avoid collisions caused by multiple OBUs sending information simultaneously. On account of the intricate attributes of the contention mechanism among the transmissions of mobile OBUs, an approximate model introduced in [27] is adopted to calculate the competition delay $t_{c}$ when an OBU at road point *s* in trajectory updates data to a RSU $N_{i}$, as follows:

(19) $$t_{c}=\frac{\bar{\xi_{1}}}{1-P^{tr}}$$

where $P^{tr}$ refers to the probability of generating at least a single update by other competition OBUs between the double consecutive back-off counter states of an OBU, ${\bar{\xi }}_{1}$ is an intermediate parameter, *s* is the center road point of the discretized road segment. These two parameters are calculated as follows:

(20) $$P^{tr}=1-{\left(\frac{N_{C}-1}{N_{C}+1}\right)}^{N_{M}-1}\;E\left[T_{\mathrm{trans}\;}\right]=P^{tr}\left(T_{p}+T_{DIFS}\right)+\left(1-P^{tr}\right)T_{F}\;\bar{\xi_{1}}=\left(\left(N_{C}+1\right)E\left[T_{\mathrm{trans}\;}\right]\right)/2+T_{p}$$

where $N_{C}$ is the contention window size in CSMA/CA, $N_{M}$ is the number of OBUs participating in channel competition, $T_{\mathrm{trans}}$ denotes the average time interval between the double consecutive back-off counter states, $T_{p}$ represents the time for transmitting a data packet, and it is able to be calculated as the packet size divided by the channel rate, $T_{F}$ is the maximum duration that an OBU keeps sensing idle channels before decrementing its back-off counter, and $T_{DIFS}$ is predetermined period time called distributed interframe space.

In this model, $N_{C}$, $T_{trans}$, $T_{p}$, and $T_{F}$ configured by the hardware and communication protocol are all fixed parameters. Besides, the number $N_{M}$ of OBUs participating in channel competition with an OBU, when the OBU is at point s in the trajectory and updating data to the corresponding RSU $N_{i}$, has a significant impact on $t_{c}$, which is calculated as follows:

(21) $$N_{M}=\sum_{j=1}^{W}l_{j}^{\prime }\rho$$

where *W* is the quantity of all trajectories in *Φ*, $l_{j}^{\prime }$ is the part of trajectory $L_{j}$ covered by the RSU $N_{i},j\in \{1,2,\ldots ,W\},i\in \{1,2,\ldots ,e\}$, *ρ* is the average number of mobile OBUs per unit length in trajectories. Specifically, if the deployed RSU $N_{i}$ covers all points in $l_{j}^{\prime }$, and the points $s_{i}\in L_{j}\backslash l_{j}^{\prime }$ is not covered by $r_{j}$, then $l_{j}^{\prime }$ is the part of trajectory $L_{j}$ covered by the RSU $N_{i}$.

It should be pointed out that all covered points can be identified after the deployment of RSUs, and then the competition delay $t_{c}$ can be calculated when a mobile OBU at position *s* updates data to its nearest RSU $N_{i}$.

#### 3.3.2. Processing Time

The processing time $t_{p}$ required for the RSU $N_{i}$ to complete all of the assigned tasks is denoted as follows:

(22) $$t_{p}\left(N_{i}\right)=\sum_{T_{k}^{i}\in TaskN_{i}}PT\left(T_{k}^{i}\right)=\frac{\sum_{T_{k}^{i}\in TaskN_{i}\;}\;\mathrm{length}\;\left(T_{k}\right)}{\;\mathrm{CPUrate}\;\left(N_{i}\right)}$$

where $PT\left(T_{k}^{i}\right)$ is the time for the RSU $N_{i}$ to process $T_{k},\;\mathrm{length}\;\left(T_{k}\right)$ is the instruction amounts of $T_{k}$, and CPUrate ($N_{i}$) represents the CPU calculation rate of RSU $N_{i}$, which is determined by the clock rate, core quantity of processors, instruction level parallelism, etc.

Therefore, the execution time of the *i*th RSU $N_{i}$ can be calculated as follows:

(23) $$G_{N_{i}}=t_{c}\left(N_{i}\right)+t_{p}\left(N_{i}\right)$$

*Total Time* is the whole time required for the IoV system to fulfill all operations. It is defined as the moment from sending the request to the moment when the last task is completed, which equals to the maximum period of time spent by RSUs. *Total Time* is determined as follows:

(24) $$\mathrm{Total}\;\mathrm{Time}={\mathrm{Max}}_{1\leq i\leq e}\left[G_{N_{i}}\right]$$

#### 3.3.3. Calculating Cost Model

When a task is calculated by a RSU, a certain amount of costs for processing, memory usage, and bandwidth usage are supposed to be considered. The estimated cost of fulfilling task $T_{k}$ by RSU $N_{i}$ is expressed as:

(25) $$Z_{T_{k}^{i}}=c_{p}\left(T_{k}^{i}\right)+c_{m}\left(T_{k}^{i}\right)+c_{b}\left(T_{k}^{i}\right)$$

Besides, the processing cost is defined as follows:

(26) $$c_{p}\left(T_{k}^{i}\right)=c_{1}*\mathrm{PT}\;\left(T_{k}^{i}\right)$$

where $c_{1}$ refers to the usage of the CPU cost per time unit in the RSU $N_{i}$, *PT* ($T_{k}$) is defined as shown in Equation (22), $c_{2}$ means the cost of memory usage with per data unit in the RSU $N_{i}$, and Mem ($T_{k}$) memory used by Task $T_{k}$. The cost of memory usage is:

(27) $$c_{m}\left(T_{k}^{i}\right)=c_{2}*\mathrm{Mem}\left(T_{k}^{i}\right)$$

The task $T_{k}$ processed in the RSU $N_{i}$ requires a certain bandwidth (*Bw)* ($T_{k}$), which is the sum of the size of input files and output files. In addition, the bandwidth usage cost per data unit is denoted as $c_{3}$, and the bandwidth usage cost is represented as below:

(28) $$c_{b}\left(T_{k}^{i}\right)=c_{3}*Bw\left(T_{k}^{i}\right)$$

The calculating cost of the total tasks completed within the IoV system is represented:

(29) $$\mathrm{Total}\;\mathrm{Cost}\;=\sum_{T_{k}^{i}\in \;\mathrm{TaskScheduling}\;}Z_{T_{k}^{i}}$$

### 3.4. Reliability Model

In such a complex network environment, the task scheduling of multiple workflow applications is a NP-hard process [28]. Reliability is an indispensable constraint of the scheduling algorithm to ensure that the QoS of workflow applications is within the tolerable range. Reliability is synonymous with “guarantee”, which is a term widely used in the International Telecommunication Union (ITU) [3]. Packet loss rate refers to the probability that the receiver loses the packets it should receive under certain coverage and channel quality conditions. Generally, the packet loss rate can bring disastrous consequences to vehicle driving safety in vehicle-to-everything (V2X) communication. However, few literatures introduce the factor of packet loss rate caused by occlusion into the definition of reliability [29].

In the scenario we proposed, the dense dynamic traffic flow on lane 1 will lead to the instability of the received signal, thus causing the loss of signal packets. It is widely recognized that the signal becomes useless when the packet loss rate of the signal is less than a threshold in signal propagation [30]. Reliability in V2X communication is supposed to meet the following requirements: packet loss rate is less than *ϕ* within the safe braking distance. As mentioned above, reliability is modeled as a constraint on the average packet loss rate:

(30) $$P_{loss\;}^{s}\leq \phi$$

where ϕ is the threshold of the average packet loss rate. This inequality ensures that the average packet loss rate is bounded, thus constraining the reliability of the wireless link. For each task, *α* can be calculated by the relative position of the OBU and the corresponding RSU, $P_{loss}^{s}$ can be calculated according to Equation (14).

### 3.5. Optimization Target Model

The target of our research is to optimize the execution time and calculate the costs of all tasks under the constraint of reliability. In this task, scheduling issue turns is classified as a multi-objective optimization problem:

(31) $$\;\min\;\mathrm{Total}\;\mathrm{Time},\;\mathrm{Total}\;\mathrm{Cost}\;\mathrm{subject}\;\mathrm{to}\;:\sum_{i=1}^{e}p_{i}=1\;P_{loss\;}^{s}\leq \phi ,\forall s\in CPs\;$$

where $p_{i}$ represents the coverage ratio for each RSU $N_{i}$ and $p_{i}=\frac{C_{i}}{\left|CPs\right|}$, $C_{i}$ is the amount of covered discrete road segments by $N_{i}$, *CPs* represents the whole set of discrete road segments, *s* is the central point of a road segment (road point), and s∈CPs.

## 4. Solutions

### 4.1. Preliminary Statement

To solve the scheduling issue, some scholars apply the linear weighting strategy to transform the multi-objective optimization issue into a single-objective optimization issue, and apply heuristic algorithms, such as GA, PSO, and ABC etc. to solve it [31,32]. However, the traditional GA method uses a simple, fixed evolutionary strategy, which has some defects in solving multi-objective problems. Based on this, a few scholars pay attention to the multi-objective optimization methods with the characteristics of multi direction and global search [7]. The Non-dominated Sorting Genetic Algorithm (NSGA), as a recent multi-objective genetic algorithm, performs well in the multi-objective optimization of three or more chromosomes due to its unique selection process. Furthermore, NSGA overcomes the defects of GA, such as the difficulty to determine the linear weight of single objective optimization, the disunity of dimensions among objectives, and the poor robustness [33]. Inspired by the relevant work, we propose two approximate solution methods to solve this NP-Hard problem. The first method is the GA with linear weighting method, which sets weights to change the multi-objective issue into a single-objective issue according to the sorting of importance. First, a utility function is defined as follows:

(32) $$F=\zeta *\;\mathrm{Total}\;\mathrm{Time}\;+\left(1-\zeta \right)*\;\mathrm{Total}\;\mathrm{Cost}\;$$

where ζ∈[0,1] refers to the balance coefficient between the execution time and calculating cost. For example, ζ=0.5 indicates that time has the same priority as cost in optimization. Furthermore, the system focuses on minimizing the Total Time when ζ>0.5, which indicates that passengers are willing to pay more for better performance. On the contrary, passengers pay more attention to the cost than the time when ζ<0.5.

As for the second method, TSARC is proposed based on the Non-dominated Sorting Genetic Algorithm-III (NSGA-III), and some improvements are added to it for better performance. In this method, the two variables are optimized at the same time under the constraint of reliability.

### 4.2. GA Solution

#### 4.2.1. Chromosome Coding

Each workflow application’s $WA_{j}$ is coded as a chromosome. Each gene in the chromosome corresponds to a task in the workflow application, and the value of the gene corresponds to the scheduling strategy for each task represented as $T_{k}^{i}$. For chromosome coding, the *n*-dimensional array is adopted to denote *n* genes and the sequence value represents the number of tasks. In addition, every gene contains an integer value of *k* within the range of [1, *e*], which means that the relevant task is allocated to the RSU *k*. The scheduling strategy for a group of 10 tasks is denoted as:

(33) $$\;\mathrm{Task}\;\mathrm{Scheduling}\;=\left\{T_{1}^{3},T_{2}^{1},T_{3}^{3},T_{4}^{2},T_{5}^{1},T_{6}^{2},T_{7}^{2},T_{8}^{3},T_{9}^{1},T_{10}^{3}\right\}$$

The solution represented by the chromosome is shown as follows:

(34) $$\mathrm{Chromosome}\;=\left[3,1,3,2,1,2,2,3,1,3\right]$$

where RSU 1 processes task set {2,5,9}, task set {4,6,7} is allocated to RSU 2, and RSU 3 is responsible for task set {1,3,8,10}. The reason to adopt this chromosome coding method is that it can flexibly complete genetic operations, such as crossover or mutation, create new individuals in the search space for exploring more solutions, and inherit high-quality gene fragments from parents at the same time. In addition, the task processing order is not taken into account, because the total execution time and calculation cost for completing a certain amount of tasks may not be affected by it.

#### 4.2.2. Initialization of Population

The initial population denotes the initial set of the total individuals in GA that is aimed to find the optimal solution. *N* chromosomes (*N* individuals) are generated after the coding of *N* workflow applications is completed, which forms a population of size *N*. Next, *N* individuals are randomly initialized so that more regions are found in the search space and the diversity of the first-generation population is guaranteed. Then individuals are selected within the initial population, and a few operations are performed on them to construct the next generation.

#### 4.2.3. Fitness Function

The fitness function is used to evaluate the quality of individuals in the population. Individuals with high fitness values represent solutions with good qualities. The fitness value of an individual is calculated with the utility function *F* defined in Equation (32). According to the fitness value, individuals may either survive or be eliminated from every generation.

#### 4.2.4. Genetic Operators

- Crossover Operator

**Figure 3:**
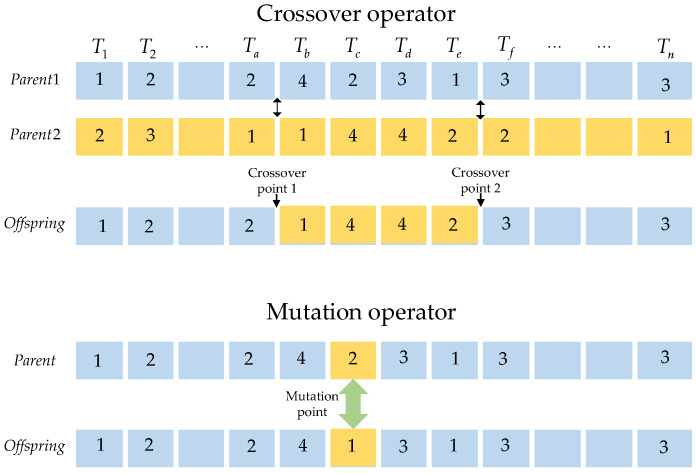
The operators of chromosome crossover and mutation.

The chromosome code is considered as an array with integers, and the cross operator between two points is used to generate offspring that inherit excellent genes from parents. The crossover operators are shown in Figure 3.

In the crossover operator, two intersections are selected randomly. The first parent exchanges the segment of the gene with the second one, and the other genes are retained to generate a new individual. In the process of population crossover, parent selection will affect the efficiency of the algorithm. The crossover rate of each individual is *ζ*. Moreover, every individual within the population is considered as the first parent with a probability of *ζ*. The second parent is selected to participate in the crossover processing by applying the roulette selection method, which ensures individuals with high fitness values to be selected.

b. Selection Operator

After the operators of crossover and mutation, natural selection is conducted immediately. Then the fitness value of offspring was calculated and compared with that of parents. If the offspring is superior to the parents, the individuals will be retained to build up the next generation population. On the contrary, the offspring will be discarded and then the parent will remain in the population.

c. Mutation Operator

Mutation is a process that reduces the limitation of the crossover operator. After the selection operator, the *N* individuals form a new population. Every individual takes part in the mutation of a point with a rate of *η*. The locations of the mutation are randomly generated and replaced with different values of the genes, as shown in Figure 3.

The pseudocode of the execution time and calculating cost is shown in Algorithm 1, and the pseudocode of GA method in Algorithm 2:
**Algorithm 1** Execution time and calculating cost of the *j*th workflow application**Input**: $WA_{j}$ **Output**: TotalTime, TotalCost 1. TotalTime=0, TotalCost=0; 2. **for** *j* = 1 to *u* do 3.  Calculate execution time according to Equation (24); 4.  Calculate calculating cost according to Equation (29); 5. **end for** 6. **return**
TotalTime, TotalCost


**Algorithm 2** GA Method
**Input**: $WA_{j}$ **Output**: TaskScheduling 1: Initialize the gene value $T_{k}^{i}$ of the first-generation population; 2: **While**
n≤N
**do** 3: **for**
j=1
**to** e 4:  $\left(TotalTime,Total\mathrm{Cost})=algorithm1(WA_{j}\right)$; 5:  Calculate *F*($WA_{j}$); 6:  $W{A_{j}}^{\prime }$= Roulette ($WA_{j}$); 7:  **if**
$F(W{A_{j}}^{\prime })>F(WA_{j})$ 8:    $WA_{j}$ = $W{A_{j}}^{\prime }$ 9:    Intersect ($WA_{j}$), Mutate ($WA_{j}$) 10:  **else** 11:   $W{A_{j}}^{\prime }$= Roulette ($WA_{j}$) 12:  **end**
13: **end for** 14: t=t+1*,*
n=n+1 15: **end while**
16: $P_{loss}^{s}\leq \phi$ 17: **end for** 18: **return**
TaskScheduling


### 4.3. TSARC Solution

#### 4.3.1. Structure of TSARC

TSARC is a multi-objective optimization algorithm based on reference points and reference vectors. Furthermore, each task consists of some sub modules, that is, time module, cost module, position module, and the basic attributes, which are stored in the form of structures. In addition, the part of the competition delay in the execution time is calculated by Equation (19). The scheduling of all workflow applications must meet the constraints of reliability, which is based on the real-time relative position of the OBU and RSU. After the coding operator in Section 4.2.1 for each application is completed, TSARC runs on the MEC broker to obtain the corresponding scheduling method for each task.

#### 4.3.2. Main Steps of TSARC

- Coding and Initialization

Before running the TSARC algorithm, the workflow application needs to be coded first. The coding process is the same as Section 4.2.1. After the coding operator, the values of all genes in the chromosomes are randomly initialized within [0, *e*]. Then genetic operators are applied to population *A* to form a new population *B*, which includes operators of selection, mutation, and crossover. Finally, the two populations *A* and *B* are combined to form a new population *C* with a size of 2*N*.

b. Quick Non-Dominated Sorting

First, the individuals dominated by individual p in the population form a set $S_{p}$, and the dominated chromosomes are counted in $n_{p}$. Next, the individuals that satisfy $n_{p}=0$ form the first frontal surface $F_{1}$, and the *rank* of these individuals is recorded as 1. For the ith frontal surface, the set $S_{p}$ composed of individuals dominated by individual *p* is selected. Let $n_{p}=n_{p}-1$, if $n_{p}==0$ satisfy at this time, then these individuals will consist of the (*i* + 1)th frontal surface $F_{i+1}$. After the non-dominated sorting, individuals with a *rank* of 1,2,3… are selected for the purpose of constructing a new population *D*, and the sorting is stopped until the population size reaches *N*.

c. Adaptive Normalization of Population Members

First, the minimum value of each dimension i in the function of multi-objective optimization is calculated, that is, the minimum value of execution time and calculating cost. The minimum value of the ith dimension is $Z_{i}$, which is the ideal point, as mentioned in [34]. The first quantization equation is shown as follows:

(35) $$f_{i}^{\prime }\left(x\right)=f_{i}\left(x\right)-z_{i}^{min}$$

Next, the extreme point is calculated by the achievement scalarizing function (ASF) on each dimension. The ASF function is shown as follows:

(36) $$ASF\left(X,W\right)=\underset{i=1:u}{max}\frac{f_{i}^{\prime }\left(x\right)}{W_{i}}$$

Finally, all functions are traversed to find the individual with the minimum value of ASF, that is, the extreme point. Then, their intercept on the corresponding coordinate axis according to the specific function value of these points is calculated and recorded as $a_{i}$. After the value of $Z_{i}$ and $a_{i}$ is obtained, the result of adaptive normalization is recorded as $f_{i}^{n}$, which can be calculated as follows:

(37) $$f_{i}^{n}=\frac{f_{i}^{\prime }\left(x\right)}{a_{i}}$$

d. Associate Individuals and Reference Points

**Figure 4:**
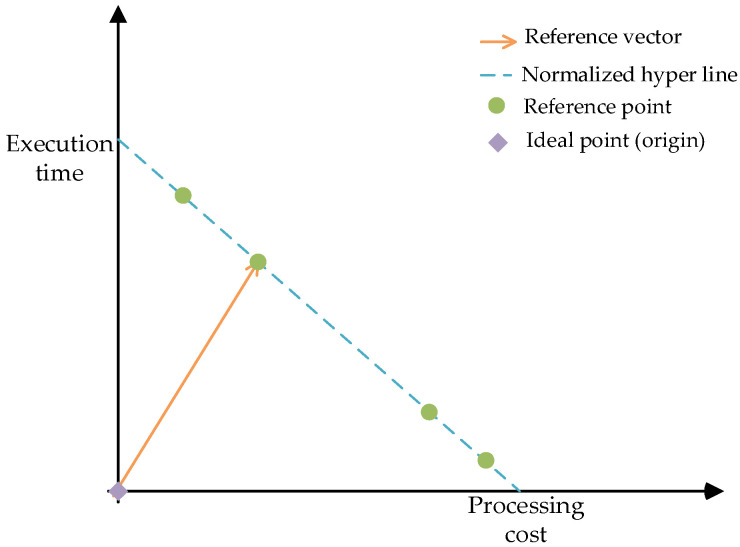
The reference points of TSARC.

Based on the normalization operation, the reference point of TSARC can be obtained, as shown in Figure 4. 

These reference points are descending in ascending order and do not need to be evaluated in each generation after recursive calculation. These reference points are connected to constitute a normalized hyper line [34]. Furthermore, a reference vector that connects the ideal point (origin) and the reference point is established. TSARC is an algorithm based on reference vectors, so it traverses all reference vectors to find the nearest reference point to each individual in each population. Based on this, the quantity of related individuals of a reference point is arbitrary.

e. Selection of Reference Point and Next Generation Population

After the non-dominated sorting process, it is supposed that the total quantity of chromosomes from the first frontal surface to the *L*th frontal surface is supposed to exceed the population size *N* for the first time. Next, $S_{t+1}$ is defined as a set that includes all individuals in $F_{L}$. Because the size of $S_{t+1}$ exceeds the preset number of chromosomes, the corresponding selection of the next generation population is required. The steps of the selection process are as follows: the first step of selection process is to traverse each reference point, and determine how many times it is referenced by $S_{t+1}$ that excludes $F_{L}$. Then, the reference point with the least references is found, that is, the reference point associated with the least individuals. Finally, the quantity of references is recorded as $p_{l}$.

- If $P_{j}==0$ is true and there is an individual related to this reference vector in the frontal surface $F_{L}$, the point with the smallest distance should be found and extracted. Then, it should be added to the next generation individuals, and $p_{l}=p_{l}+1$ is set.
- If there is no individual associated with the reference points in the frontal surface $F_{L}$, the reference vector should be deleted. If $p_{j}>0$ satisfies, its nearest reference point should be selected and the process of cycling is kept until the population size reaches *N*.

#### 4.3.3. Pseudocode of TSARC

Based on the above introduction and analysis of TSARC, the selection process of the next generation population is presented in Algorithm 3. Furthermore, the pseudocode of TSARC is presented in Algorithm 4, which consists of Algorithms 1 and 3.
**Algorithm 3** Selection of next generation population **Input**: Previous generation population, $S_{t}$ **Output**: Next generation population, $S_{t+1}$ 1. Non-dominated layer $F_{L}$= Non-dominated sorting ($S_{t}$); 2.$P_{t}=F_{1}+F_{2}+\ldots +F_{L}$; 3. **if** $Size(P_{t})<N_{pop}$ 4.  Normalization based on Equations (35)–(37); 5.  Obtain the super connector based on the reference vector in Figure 4; 6.  $F_{L}=F_{L}+z$; 7.  $S_{t+1}=S_{t}\cup F_{L}$; 8. **else** 9. $S_{t+1}=P_{t}$; 10. **end for** 11. **return** $S_{t+1}$

**Algorithm 4** TSARC Method**Input**: The size *N* of population $S_{0}$, all workflow applications $WA_{j}$ **Output**:TaskScheduling 1: Initialize the gene value $T_{k}^{i}$ of the first-generation population; 2: $S_{t}=S_{0}$; 3: **while** n≤N
**do** 4:   ** for j=1 to** e 5:    Intersect ($WA_{i}$), Mutate ($WA_{i}$); 6:    $\left(TotalTime,TotalCost)=algorithm1(WA_{j}\right)$; 7:   **end for** 8: The next generation population $S_{t+1}=algorithm3(S_{t})$; 9: t=t+1*,*
n=n+1; 10: **end while**
11: $P_{loss}^{s}\leq \phi$; 12: **return TaskScheduling**


## 5. Numerical Results

This chapter first defines the values of key parameters and simulates a road topology with a dynamic dense traffic flow. Next, the three methods are evaluated via numerical simulation. In the part of the experiment, NSGA-II is added as the baseline algorithm to compare with GA and TSARC. NSGA-II is one of the most popular multi-objective genetic algorithms for optimization. It reduces the complexity of non-inferior sorting genetic algorithms, has the advantages of a fast-running speed, and a good convergence of the solution set, and becomes the benchmark of other multi-objective optimization algorithms. Finally, the experimental results are described and analyzed.

### 5.1. Simulation Settings

**Figure 5:**
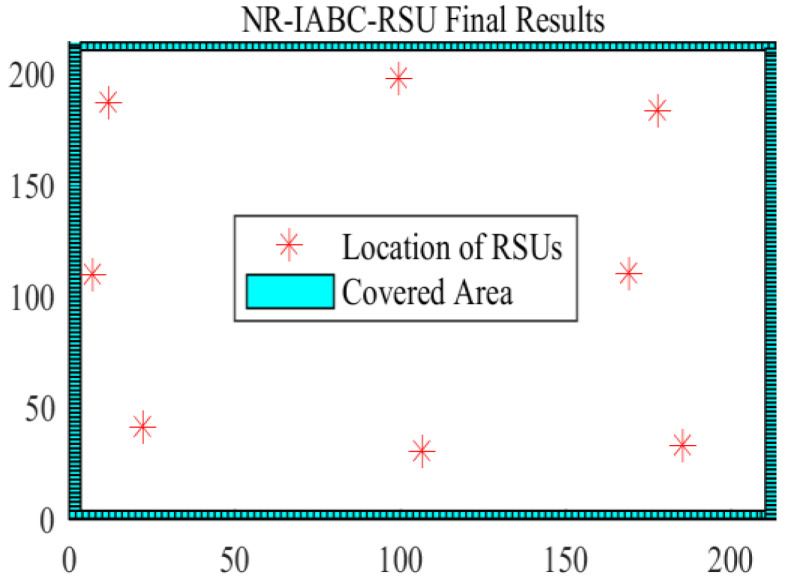
The full coverage deployment diagram of eight RSUs.

By using the RSU deploy method proposed in our previous work, an RSU layer consisting of eight RSUs is deployed by the NR-IABC algorithm [12], which achieved full coverage of the four target roads. The full coverage deployment diagram of the eight RSUs is shown in Figure 5.

Next, 20~200 tasks are generated randomly. For each task, it contains the quantity and size of signal packets, the location of the source OBU and other relevant information. In addition, the quantity of the packet is randomly generated and the size of each signal packet is fixed to be 300 bytes. Each RSU has a certain processing capacity, which is expressed by the processing rate (MIPS (millions of instructions per second)), along with the cost of the CPU, bandwidth, and memory [32]. The attribute parameters of tasks and CPUs are shown in Table 3. The traffic flow density on each road and the real-time position of each vehicle are generated randomly. For the purpose of creating a real-life scene, the range of values refer to the dataset, the British Highway Traffic Flow Data Set [35], which counts the number of vehicles that travel past the count point on an average day of the year (by direction of travel). Furthermore, the range of the traffic flow density is set to be 0~1 vehicles/s and the number of vehicles on the four roads is generated with 24 as the maximum quantity of vehicles that can be carried on the road. For the constraint of the packet loss rate threshold *ϕ* in the reliability constraint, we referred to the application parameters in the actual engineering situation and set its value to be 10% [36]. Finally, a population that concluded 100 individuals is initialized. In addition, the number of iterations is 200, and the balance coefficient is set to be ζ=0.5. The parameters used in the simulation experiment are summarized in Table 3. As for other parameter settings, they can be found in our previous work [12]. All numerical analysis is generated based on the mean of 50 independent simulation results to avoid the influence of random factors. The simulations are carried out on a computer with AMD Ryzen 7 5800H CPU, using MATLAB version of R2016a.

### 5.2. Result Analysis

#### 5.2.1. Comparison of Execution Time

**Figure 6:**
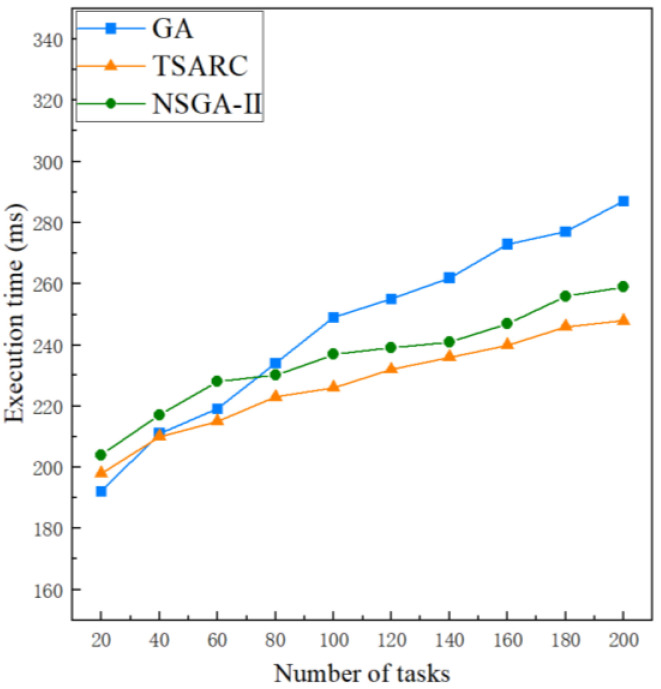
Comparison of the execution time of the three methods under different number of tasks.

Figure 6 shows the execution time comparison of the three methods under the same setting (γ=0.1,ζ=0.5). It is obvious that the execution times of the three methods increase with the growth of the number of tasks *n*. It can be observed that TSARC is not the best method for small-scale tasks (n≤40) and GA performs best. Furthermore, TSARC outperforms GA when n>40 and the disparity between them expands gradually with the growth of *n*. The reason is that the search space increases at the same time when *n* increases. In this condition, GA cannot converge in limited iterations, and it cannot explore the entire space with limited individuals, resulting in a longer convergence time. However, TSARC has a good convergence speed for large-scale tasks by using fast non-dominated sorting. Basically, TSARC can save 10% to 15% of execution time in large-scale tasks in contrast to GA. In addition, the performance of TSARC is better than that of NSGA-II in all cases. The reason is that TSARC maintains the diversity of the population, so it can obtain more optimal solutions than NSGA-II. Besides, the performance of TSARC is always more stable than GA and NSGA-II. Generally, the execution time is regarded as one of the key parameters of QoS. Therefore, some passengers are willing to spend more money to experience higher performance services due to the demand for QoS.

#### 5.2.2. Comparison of Calculating Cost

**Figure 7:**
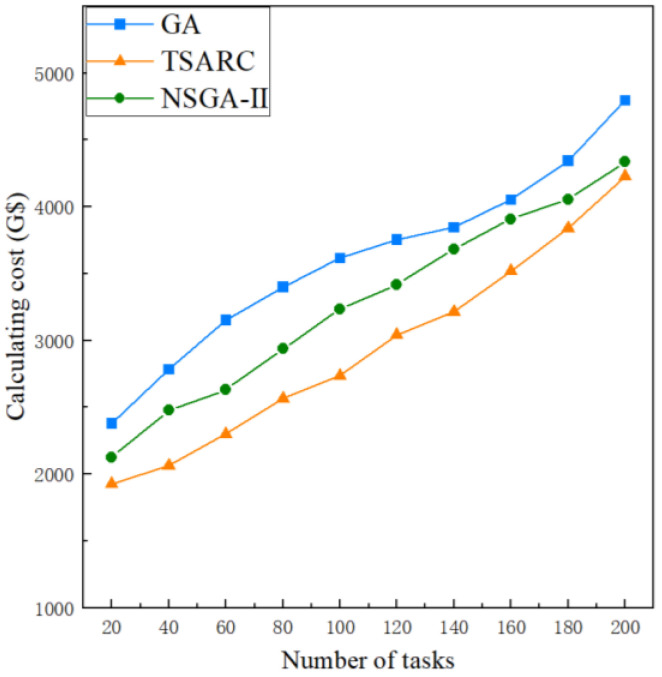
The comparison of calculating cost of the three methods under different number of tasks.

To illustrate the effect of *n* on the calculating cost, *n* is set to be 20 to 200, respectively. The impact of *n* under the same setting (γ=0.1,ζ=0.5) is shown in Figure 7. It can be observed that the calculating cost of these methods all increase with the growth of n and the increasing trend of calculating cost slows down gradually. Obviously, TSARC outperforms GA and NSGA-II in all cases because less cost is needed to complete the same tasks, which can generally save 15% to 25%. When compared with GA, TSARC uses an elite strategy to store all individuals in the population hierarchically and sort them according to the degree of crowding. It retains the excellent individuals in the parent population and is less likely to lose the best individuals than the roulette selection method adopted by GA, which indicates that it has a better convergence accuracy and reduces the selection pressure. When compared with NSGA-II, the selection mechanism of TSARC is different from that of NSGA-II. NSGA-II uses the crowding distance and crowding degree to select individuals at the same non-dominated level, while TSARC uses distribution reference points to maintain population diversity under high-dimensional targets. When optimizing the execution time and calculation cost at the same time, the convergence and diversity of NSGA-II become worse if the crowding distance is used, and it is easy to fall into the local optimum. Therefore, TSARC has the best cost-effectiveness among the three methods. Especially, when the value of *n* is large enough, good cost-effectiveness can mitigate the impact of the lack of computing resources.

#### 5.2.3. Analysis of the Influence of Traffic Flow Densities

**Figure 8:**
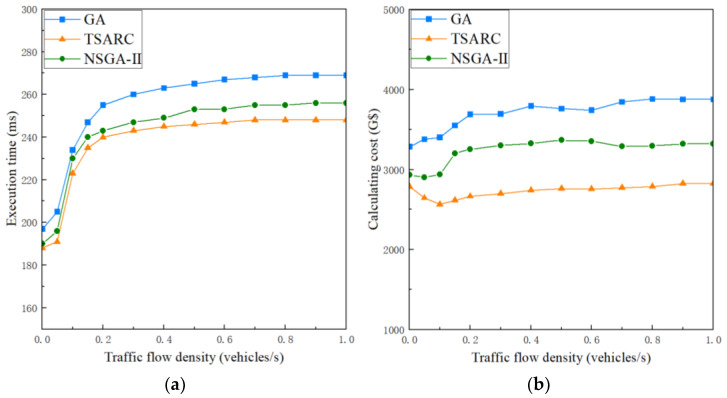
The influence of different traffic flow density on the execution time and calculating cost of the three methods: (**a**) execution time; (**b**) calculating cost.

The traffic flow density is able to have an effect on the average packet loss rate, and then further affect the strategy of task scheduling. We discuss the influence of traffic flow density *γ* on the execution time and calculating cost of the three methods under the same setting (n=80,ζ=0.5), as shown in Figure 8a and Figure 8b, respectively. When γ<0.2, the execution time and calculating cost increase significantly with the growth of *γ*. When γ≥0.2, the growth rate of the three methods gradually slows down, which indicates that the occlusion effect tends to be saturated. The execution time and calculating cost both maintain a fixed value under the condition of γ>0.9, which means that the packet loss rate exceeds *ϕ*. According to Equations (14) and (30), when the packet loss rate $P_{loss}$ exceeds *ϕ*, the communication link is always in an interrupted state no matter what the value of *γ* is. Figure 8a shows that the execution time of TSARC takes the first place in each dataset, which is 7.81% and 3.13% better than GA and NSGA-II, respectively. In Figure 8b, it is obvious that the TSARC method shows high cost-effectiveness in almost all datasets, saving 27.05% and 14.09% when compared with GA and NSGA-II, respectively. In addition, TSARC and NSGA-II have a temporary decline when γ<0.2. This is because they tend to migrate tasks to cheaper RSUs for execution to ease pressures on resources. Furthermore, it can be observed that the curve of TSARC is smoother than that of GA and NSGA-II, which shows better stability in solving the multiple objective optimization problem. 

#### 5.2.4. Analysis of the Influence of Balance Coefficients

**Figure 9:**
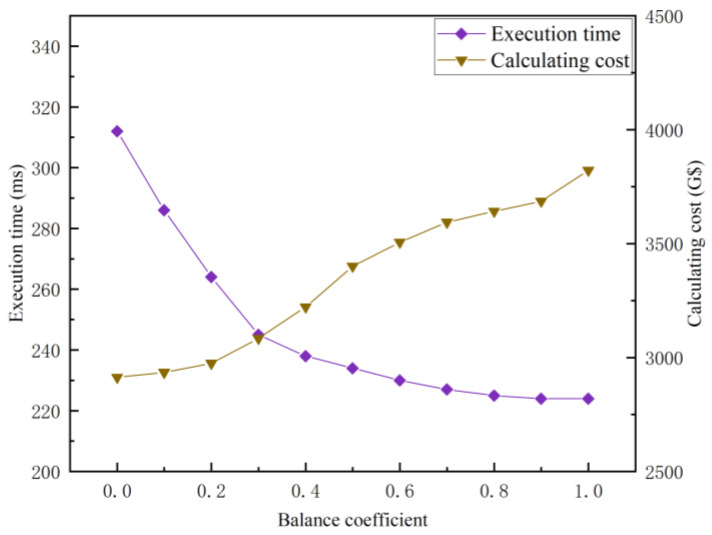
The influence of different balance coefficients on the execution time and calculating cost of the GA method.

Setting different balance coefficients will affect the performance of GA. Figure 9 shows the influence of the balance coefficient under the same setting (n=80,γ=0.1) on the GA method. The calculating cost is the maximum in the case of ζ=0, while the execution time is the minimum, which indicates that the tasks are centrally allocated to the cheapest RSUs. When ζ=0.5, the calculating cost increased by 16.75% and the execution time decreased by 25.00%, corresponding to the condition of ζ=0. When ζ gradually increases to 1, the execution time decreased slightly together with a small amount of cost added, which shows that the utility function concentrates on the optimization of the calculating cost. The series of experiments shows that the GA method may flexibly meet the users’ requirements for the execution time or calculation cost by adjusting ζ dynamically. However, the balance coefficient is preset in reality, so it is inconvenient to adaptively meet the diversified QoS. Therefore, TSARC shows better robustness than GA for dense traffic flow scenarios.

#### 5.2.5. Comparison of Solving Efficiency of the Three Methods 

**Figure 10:**
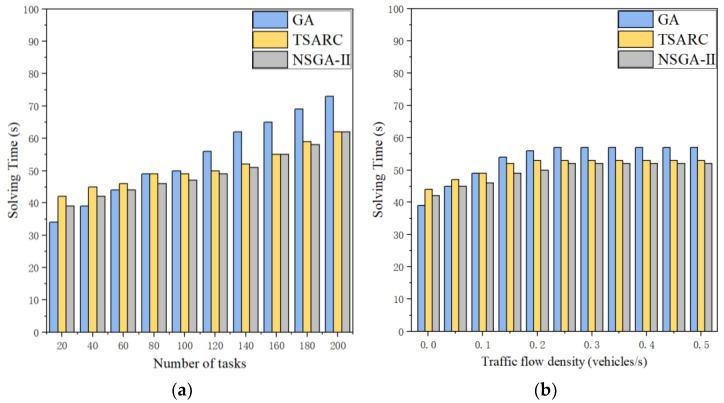
Comparison of the efficiency of the three methods under different parameters: (**a**) number of tasks (*γ* = 0.1); (**b**) traffic flow density (*n* = 80).

The solving efficiency of the three methods is compared in Figure 10. It is obvious that the solving time of them all increases with the growth of the number of tasks in large-scale and small-scale datasets. Basically, GA does have a higher solving efficiency than TSARC in small-scale datasets, but TSARC performs more steadily. When the number of tasks exceed 80, TSARC has a higher solving efficiency than GA, which can generally save 15%~20% of solving time. In Figure 10b, it can be observed that the solving time of the three methods gradually increases when *γ* increases under the condition of γ≤0.2. Then they tend to a stable approximate value of 53 when γ>0.2. In addition, although NSGA-II has the highest solving efficiency, the disparity between NSGA-II and TSARC is negligible. Combining the results of Figure 10a,b, it can be derived that our proposed method is not the best on small-scale datasets, and GA takes the first place among them. In addition, NSGA-II shows its advantages in solving efficiency on large-scale datasets, but TSARC is almost equal to it.

## 6. Conclusions

In an IoV system with limited wireless and computing resources, the workflow applications on it require high QoS for passengers. However, the real-time task scheduling of RSUs is faced with a series of difficulties, such as dynamic occlusion, bandwidth limitation, and obvious delay. The high mobility of vehicles also makes the V2I communication become intractable. In addition, there must be redundant coverage in the full coverage model of RSUs, and the upload paths of OBUs also become diversified. Therefore, it is a great challenge to formulate a proper task scheduling method under the above conditions. Besides, the reliability of the task scheduling algorithm is a constraint index that should be considered to evaluate the wireless link. The task scheduling algorithm proposed in this paper is implemented in a V2I communication architecture with dynamic dense traffic flow. Firstly, a traffic flow model with dynamic occlusion is established. Then, the reliability is redefined based on the constraint of average packet loss. Next, TSARC is proposed to simultaneously optimize the execution time and the calculating cost of workflow applications, which can be clarified as a multi-objective optimization problem. The algorithm is based on the improved NSGA-III algorithm, where the Pareto optimal solution of time and cost is found by constructing reference points and reference vectors. Finally, the experiment discusses the influence of the key parameters and the comparison with two other algorithms. Numerical results show that the TSARC method has better scalability and efficiency than the other methods. In the future work, RSU deployment and resource scheduling models will be further considered jointly.

## Figures and Tables

**Table 1:** Variables and parameters required in the problem statement.

Parameters	Description
$s_{0}$	Road width
*γ*	Truck arrival rate
*λ*	The wavelength of V2X signal
$R_{1}$	The radius of the first Fresnel ellipsoid
d	The occlusion length of traffic flow
w	Reflection coefficient
v	Kirchhoff parameter
*f*	The frequency of V2X signal
*τ*	Minimum received sensitivity threshold
$D_{s}$	The transmitting power of the OBU
$D_{r}$	The received power of the RSU
$T_{DIFS}$	Distributed inter-frame spacing
$T_{F}$	The length of the competitive timeslot
$T_{P}$	The uploading duration of the signal packet
$B_{R}$	The rate of the wireless channel
$B_{L}$	The size of the signal packet
*e*	The quantity of RSUs
*u*	The quantity of OBUs
*n*	The quantity of tasks
Variables	Description
*α*	A continuous variable. The included angle between AB and A’B
*θ*	A continuous variable. The elevation angle between OBU and RSU
J	A continuous variable. Diffraction attenuation
PL	A continuous variable. The attenuation in free-space path loss condition
$LOSS_{NLOS/LOS}$	A continuous variable. The attenuation in NLOS/LOS condition
$P_{NLOS/LOS}$	A continuous variable. The probability of NLOS/LOS condition
$P_{nloss}^{NLOS/LOS}$	A discrete binary variable. Whether the V2X signal is transmitted normally in NLOS/LOS condition
$P_{nloss}^{s}$	A continuous variable. The packet success rate at position *s*
$P_{loss}^{s}$	A continuous variable. The packet loss rate at position *s*
$t_{c}$	A continuous variable. Competition delay
$t_{p}$	A continuous variable. Processing time
$G_{N_{i}}$	A continuous variable. The execution time of the *i*th RSU
TotalTime	A continuous variable. Total execution time
$C_{p}$	A continuous variable. Processing cost
$C_{m}$	A continuous variable. Memory usage cost
$C_{b}$	A continuous variable. Bandwidth usage cost
$Z_{T_{k}^{i}}$	A continuous variable. The calculating cost of the *k*th task by the *i*th RSU
TotalCost	A continuous variable. Total calculating cost

**Table 2:** Steps of task scheduling.

Step	Operation
1	OBUs send requests to the nearest RSUs to connect to them
2	These requests are forwarded to the MEC broker for analysis
3	Each workflow application is decomposed into a group of tasks
4	The number of instructions and the required resource usage are estimated
5	The MEC broker runs the task scheduling algorithm
6	The tasks are assigned to the corresponding RSUs
7	Each RSU handles its assigned tasks
8	All task processing results are fed back to the MEC broker
9	The MEC broker merges all of the results when all tasks are completed
10	The response is sent to the OBUs through RSUs to connect with them

**Table 3:** Simulation parameters.

Parameters	Values
$s_{0}$	3.5 (m)
*λ*	0.05 (m)
*f*	5.9 (GHz)
*τ*	−80 (dBm)
*L*	200 (m)
*MaxIt*	200
*nPop*	100
*K*	126
*ε*	15
*ϕ*	10%
$T_{DIFS}$	$1.28\times 10^{-7}(s)$
$T_{F}$	$5\times 10^{-8}(s)$
$T_{P}$	$2.4\times 10^{-3}(s)$
$B_{R}$	1 (MB/s)
$B_{L}$	300 (B)
Number	8
Rate of the CPU	[500, 1000] (MIPS)
Required memory	[50, 200] (MB)
Size of the input file	[10, 100] (MB)
Size of the output file	[10, 100] (MB)
Usage cost of the CPU	[0.1, 0.4] (G$/s)
Cost of the memory usage	[0.01, 0.03] (G$/MB)
Cost of the bandwidth usage	[0.01, 0.02] (G$/MB)
Number of instructions	$[1,\;100]\;\times \;10^{9}$ (instructions)
